# Identification and characterization of [6]-shogaol from ginger as inhibitor of vascular smooth muscle cell proliferation

**DOI:** 10.1002/mnfr.201400791

**Published:** 2015-03-16

**Authors:** Rongxia Liu, Elke H Heiss, Nadine Sider, Andreas Schinkovitz, Barbara Gröblacher, Dean Guo, Franz Bucar, Rudolf Bauer, Verena M Dirsch, Atanas G Atanasov

**Affiliations:** 1 Department of Pharmacognosy, University of ViennaVienna, Austria; 2 Shanghai Research Center for Modernization of Traditional Chinese Medicine, National Engineering Laboratory for TCM Standardization Technology, Shanghai Institute of Materia Medica, Shanghai Institutes for Biological Sciences, Chinese Academy of SciencesShanghai, P. R. China; 3 Institute of Pharmaceutical Sciences, Department of Pharmacognosy, Karl-Franzens-University GrazGraz, Austria

**Keywords:** Ginger, Heme oxygenase-1, Nrf2, [6]-Shogaol, Vascular smooth muscle cell

## Abstract

**Scope:**

Vascular smooth muscle cell (VSMC) proliferation is involved in the pathogenesis of cardiovascular disease, making the identification of new counteracting agents and their mechanisms of action relevant. Ginger and its constituents have been reported to improve cardiovascular health, but no studies exist addressing a potential interference with VSMC proliferation.

**Methods and results:**

The dichloromethane extract of ginger inhibited VSMC proliferation when monitored by resazurin metabolic conversion (IC_50_ = 2.5 μg/mL). The examination of major constituents from ginger yielded [6]-shogaol as the most active compound (IC_50_ = 2.7 μM). In the tested concentration range [6]-shogaol did not exhibit cytotoxicity toward VSMC and did not interfere with endothelial cell proliferation. [6]-shogaol inhibited DNA synthesis and induced accumulation of the VSMC in the G_0_/G_1_ cell-cycle phase accompanied with activation of the nuclear factor-erythroid 2-related factor 2 (Nrf2)/HO-1 pathway. Since [6]-shogaol lost its antiproliferative activity in the presence of the heme oxygenase-1 (HO-1) inhibitor tin protoporphyrin IX, HO-1 induction appears to contribute to the antiproliferative effect.

**Conclusion:**

This study demonstrates for the first time inhibitory potential of ginger constituents on VSMC proliferation. The presented data suggest that [6]-shogaol exerts its antiproliferative effect through accumulation of cells in the G_0_/G_1_ cell-cycle phase associated with activation of the Nrf2/HO-1 pathway.

## 1 Introduction

Ginger, *Zingiber officinale* Roscoe (Zingiberaceae), is widely used as a spice in foods and beverages, and has also a long history of use in traditional medicine for the treatment of inflammation, rheumatic disorder, indigestion, vomiting, and fever, among others 1–4. Many pharmacological activities have been reported for this plant and its major pungent principles, including anti-inflammatory, anti-tumorigenic, anti-apoptotic, anti-hyperglycemic, cancer-chemopreventive, anti-lipidemic, and anti-emetic effects 4–9.

Cardiovascular disease is the number one cause of death in the world, mainly elicited by atherosclerosis together with hypertension 10. Aberrant and accelerated vascular smooth muscle cell (VSMC) proliferation not only contributes to initial atherosclerotic plaque formation but also to restenosis (pathological renarrowing of the vessel lumen) after surgical interventions like percutaneous transluminal coronary angioplasty or bypass surgery. To overcome restenosis, drug-eluting stents have been developed, aiming at inhibiting VSMC growth by the release of antiproliferative substances. The most prominent drugs used in drug-eluting stents so far have been paclitaxel (a microtubules stabilizing agent) and sirolimus (a mTOR inhibitor). These compounds, however, exhibit a number of unresolved drug-related issues such as impaired reendothelialization and delayed thrombosis induction 11,12, which makes the discovery of novel effective compounds and molecular mechanisms suppressing VSMC proliferation highly relevant.

Plant derived natural products proved to be an excellent resource for the identification of new lead compounds 13. While ginger is reported to possess vasoprotective effects 14,15, its impact on VSMC proliferation in particular has not been studied so far. In this study, we therefore examined the antiproliferative potential of ginger extract and some of its major active components ([6]-gingerol, [6]-shogaol, zingerone, [6]-paradol, and *rac-*[6]-dihydroparadol) in VSMC, and characterized in more detail the cellular mode of action of the most active identified compound, [6]-shogaol.

## 2 Materials and methods

### 2.1 Chemicals and reagents

Primary rat aortic VSMC were purchased from Lonza (Braine-L'Alleud, Belgium) and the human umbilical vein endothelial cells (HUVECtert) were provided by Dr. Hannes Stockinger (Medical University of Vienna, Austria) 16. Wild type (WT) and isogenic nuclear factor-erythroid 2-related factor 2 (Nrf2)–/– mouse embryonic fibroblasts were kindly provided by Dr. T. Kensler, University of Pittsburgh, USA. Platelet-derived growth factors (PDGF)-BB was supplied from Bachem (Weilheim, Germany). The heme oxygenase-1 (HO-1) inhibitor tin protoporphyrin IX dichloride was from Enzo Life Sciences (Lausen, Switzerland). [6]-Parodol and *rac-*[6]-dihydroparadol were isolated as described 17. All other used reagents were from analytical grade and obtained from Sigma-Aldrich (Vienna, Austria). The anti-HO-1 antibody was from Stressgene (purchased via Enzo, Lausen, Switzerland), the anti-actin antibody [mouse anti-actin, monoclonal (Clone: C4); #69100] was from mpbio (Eschwege, Germany), and the secondary horseradish-peroxidase-coupled antibodies came from Cell Signaling (Heidelberg, Germany).

### 2.2 Ginger extraction

Root material of *Zingiber officinale* (Zingiberaceae) was purchased from PLANTASIA (Oberndorf, Austria). A voucher sample is kept at the University of Graz, Department of Pharmacogonosy under reference number 730149. Ground root material (3.17 g) was mixed with 0.97 g diatomaceous earth (Dionex Corporation, Sunnyvale, CA, USA) before being extracted with HPLC grade dichloromethane using an Accelerated Solvent Extractor (ASE 200, Dionex Corporation, Sunnyvale, CA, USA). Extraction conditions were as follows: Temperature: 44°C, cell preheating time: 1 min, pressure: 68.9 bar, static time: 5 min, flush volume: 150%. Three extraction cycles were performed yielding 168 mg of dry extract (5.3%) after solvent evaporation.

### 2.3 Cell culture

VSMC were cultivated in DMEM–F12 (1:1) supplemented with 20% fetal calf serum (FCS), 30 μg/mL gentamicin, and 15 ng/mL amphotericin B. VSMC with passage number between 6 and 14 were used in this study. HUVEC were grown in endothelial cell basal medium (EBM) supplemented with 10% FCS, 100 U/mL penicillin, 100 μg/mL streptomycin, 1% amphotericin B and EBM^TM^ SingleQuots®, containing recombinant human epidermal growth factor, hydrocortisone, gentamicin sulfate, amphotericin B and 0.4% bovine brain extract. Mouse embryonic fibroblasts (MEF) were grown in DMEM supplemented with 10% FCS, 100 U/mL penicillin, and 100 μg/mL streptomycin.

### 2.4 Resazurin conversion assay

VSMC were seeded in 96-well plates at 5 × 10^3^ cells/well. After 24 h, cells were serum-starved for 24 h to render them quiescent. Quiescent cells were pretreated for 30 min with ginger extract, compounds, or vehicle (0.1% DMSO) as indicated, and subsequently stimulated for 48 h with PDGF-BB (20 ng/mL). To measure the number of metabolically active VSMC by resazurin conversion 18,19, cells were washed with PBS and incubated in serum-free medium containing 10 μg/mL resazurin for 2 h. Total metabolic activity was measured by monitoring the increase in fluorescence at a wavelength of 590 nm using an excitation wavelength of 535 nm in a 96-well plate reader (Tecan GENios Pro). HUVECtert cells 16,20 were seeded in 96-well plates at 5 × 10^3^ cells/well. After 24 h, HUVECtert cells were treated with compounds or vehicle (0.1% DMSO) as indicated and incubated for 48 h. Then, cells were washed with PBS and incubated in culture medium containing 10 μg/mL resazurin for 2 h. The detection step is performed as described above.

### 2.5 Crystal violet biomass staining

VSMC were seeded in 96-well plates at 5 × 10^3^ cells/well. Twenty-four hours later, cells were serum starved for 24 h to render them quiescent. Quiescent cells were pretreated for 30 min with ginger extract, compounds, or vehicle (0.1% DMSO) as indicated and subsequently stimulated for 48 h with PDGF-BB (20 ng/mL). To determine the total biomass by crystal violet staining, cells were then incubated in 100 μL of crystal violet staining solution (0.5% crystal violet, 20% methanol) for 15 min and then washed with ddH_2_O. After drying, 100 μL EtOH/Na-citrate solution were added (EtOH: 0.1M Na-citrate = 1:1) and the absorbance of the samples was measured at 595 nm in a 96-well plate reader (Tecan sunrise).

### 2.6 5-Bromo-2′-deoxyuridine (BrdU) incorporation assay

VSMC were seeded in 96-well plates at 5 × 10^3^ cells/well. Twenty-four hours later, cells were serum starved for 24 h to render them quiescent. Quiescent cells were pretreated for 30 min with compounds, or vehicle (0.1% DMSO) as indicated and subsequently stimulated with PDGF-BB (20 ng/mL). To estimate de novo DNA synthesis in VSMC 21,22, BrdU was added 2 h after PDGF stimulation, and the incorporation amount was determined 22 h afterwards according to the manufacturer's instructions (Roche Diagnostics).

### 2.7 Assessment of cytotoxicity

VSMC were seeded in 96-well plates at 5 × 10^3^ cells/well. Twenty-four hours later, cells were serum starved for 24 h to render them quiescent. Quiescent cells were pretreated for 30 min with compounds, or vehicle (0.1% DMSO) as indicated, and subsequently stimulated for 24 h with PDGF-BB (20 ng/mL). Loss of cell membrane integrity as a sign for cell death can be quantified by the release of the soluble cytosolic protein lactate dehydrogenase (LDH) 20,23. For this, the supernatant of the treated cells was assessed for LDH activity. For estimation of the total LDH, identically treated samples were incubated for 45 min in the presence of 1% Triton X-100. The released and total LDH enzyme activity was measured for 30 min at the dark in the presence of 4.5 mg/mL lactate, 0.56 mg/mL NAD+, 1.69 U/mL diaphorase, 0.004% w/v BSA, 0.15% w/v sucrose, and 0.5 mM 2-p-iodophenyl-3-nitrophenyl tetrazolium chloride. The enzyme reaction was stopped with 1.78 mg/mL oxymate and the absorbance was measured at 490 nm. Potential effects on cell viability were estimated as percentage of extracellular LDH enzyme activity. The cytotoxic natural product digitonin (100 μg/mL) was used as a positive control.

### 2.8 Cell-cycle analysis

VSMC were seeded in 12-well plates at 1 × 10^4^ cells/well. Twenty-four hours later, cells were serum starved for 24 h to render them quiescent. Quiescent cells were preincubated with [6]-shogaol (10 μM) or vehicle (1% DMSO) for 30 min. PDGF-BB (20 ng/mL) was added, and 16 h later cells were trypsinized, washed once with PBS, and resuspended in a hypotonic propidium iodide (PI) solution containing 0.1% v/v Triton X-100, 0.1% w/v sodium citrate, and 50 μg/mL PI. After incubation at 4°C overnight, PI-stained nuclei were analyzed by flow cytometry (excitation 488 nm, emission 585 nm; FACScalibur; BD Biosciences, Germany).

### 2.9 Immunoblot analysis

Cells (MEF or VSMC) were seeded onto six-well plates ((3–4) × 10^5^ cells/well). Twenty-four hours later, MEF were treated with vehicle (0.1% DMSO) or [6]-shogaol at the indicated concentrations for 20 h; VSMC were serum starved for another 24 h, then quiescent cells were pretreated for 30 min with vehicle (0.1% DMSO) or [6]-shogaol as indicated and subsequently stimulated for 20 h with PDGF-BB (20 ng/mL). Then, cells were lysed and protein extracts were subjected to SDS-PAGE electrophoresis and immunoblot analysis as described 20,22. Proteins were visualized using enhanced chemiluminescence reagent and an LAS-3000 luminescent image analyzer (Fujifilm) with AIDA software (Raytest) for densitometric evaluation.

### 2.10 Cell counting

VSMC and HUVECtert were seeded and treated as described in chapter 2.4 (“Resazurin conversion assay”). Cell numbers were determined at different time points (with time point zero corresponding to the treatment with the solvent vehicle, 0.1% DMSO) upon trypan blue staining with a ViCell counter (Beckman Coulter, Brea, CA).

### 2.11 Statistical analysis

Statistical analysis was performed by analysis of variance /Bonferroni test or by *t* test (when comparing just two experimental groups). The number of experiments is given in the figure legends, and a probability value < 0.05 was considered significant. All tests were performed using GraphPad PRISM software, version 4.03.

## 3 Results

### 3.1 Ginger extract and its major bioactive components inhibit VSMC proliferation

**Figure 1:**
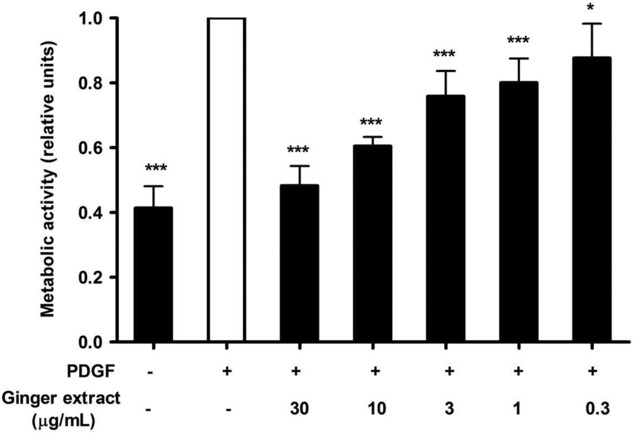
Metabolic activity of VSMC exposed to different concentrations of ginger extract. Quiescent VSMC were pretreated with the indicated concentrations of ginger dichloromethane extract or an equal volume of the solvent vehicle (0.1% DMSO) for 30 min, and then stimulated with 20 ng/mL PDGF-BB for 48 h. The metabolic activity was determined at the end of the stimulation period by the resazurin conversion method. All values are normalized to the signal obtained from the PDGF-stimulated, vehicle (0.1% DMSO)-treated cells. The data groups represent means ± SD from three independent experiments (n.s., not significant; ***p < 0.001; *p < 0.05; analysis of variance (ANOVA)/Bonferroni).

**Figure 2:**
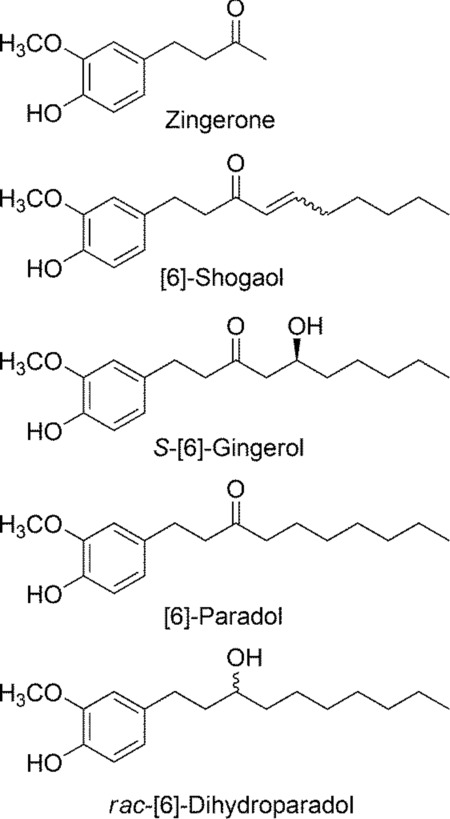
Structures of the major ginger components studied in this work.

**Figure 3:**
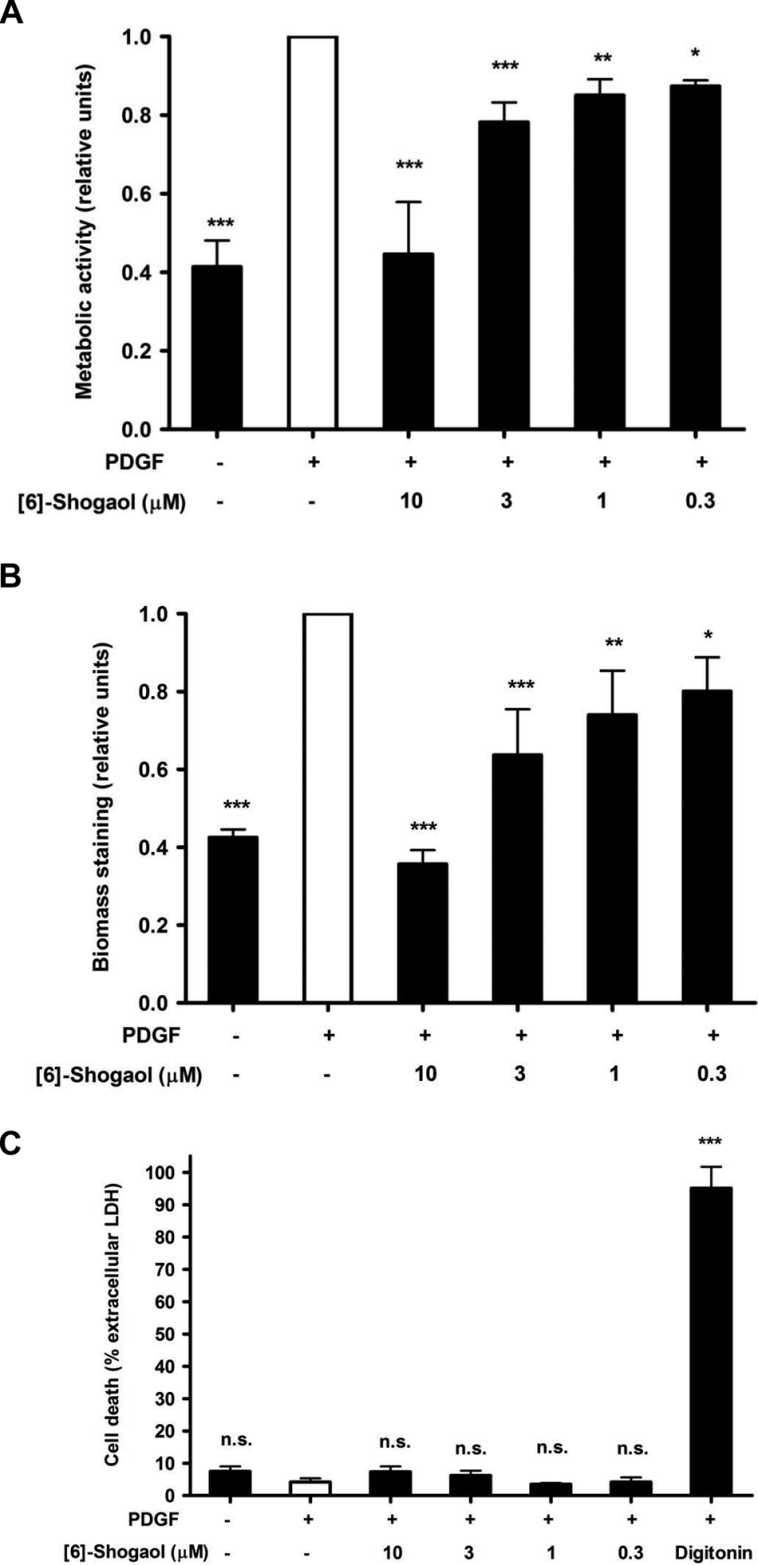
Effect of [6]-shogaol on VSMC proliferation and cell death. Quiescent VSMC were pretreated with the indicated concentrations of [6]-shogaol or an equal volume of the solvent vehicle (0.1% DMSO) for 30 min, and then stimulated with 20 ng/mL PDGF-BB. Cell proliferation was quantified by the resazurin conversion method (A) and crystal violet biomass staining (B). Cell death was estimated by measuring the cell membrane integrity through quantification of the percentage of the cytosolic enzyme lactate dehydrogenase (LDH) detected inside cells and in the extracellular medium (C). The cytotoxic natural product digitonin (100 μg/mL) was used as a positive control in the cell death assay (C). The data groups represent means ± SD from three independent experiments (n.s., not significant; ***p < 0.001; **p < 0.01; *p < 0.05; ANOVA/Bonferroni).

For evaluating whether ginger contains compounds able to inhibit PDGF-induced proliferation of VSMC, a dichloromethane extract of *Z. officinale* roots was applied at different concentrations (0.3–30 μg/mL; Fig. 1), and the total amount of metabolically active cells was measured after 48 h by the resazurin conversion method 18,19. The extract suppressed VSMC proliferation concentration dependently with an IC_50_ of 2.5 μg/mL. The highest tested concentration (30 μg/mL) decreased the signal to the basal level of the untreated growth-arrested cells (Fig. 1). To investigate which compounds from ginger are potentially mediating this antiproliferative effect, five major bioactive ginger compounds, [6]-gingerol, [6]-shogaol, zingerone, [6]-paradol, and *rac*-[6]-dihydroparadol (Fig. 2) were tested. Among these five compounds, [6]-shogaol showed best antiproliferative activity with an IC_50_ of 2.7 μM in the resazurin conversion assay (Table1 and Fig. 3A). [6]-gingerol, [6]-paradol, and *rac*-[6]-dihydroparadol, also inhibited VSMC proliferation but less potently than [6]-shogaol (with IC_50_s in the range 5.3–13.2 μM; Table1), while zingerone showed no activity up to 100 μM. The resazurin conversion method is based on the metabolic conversion of resazurin, which generally correlates well to the cell number but could be potentially sensitive to redox-active chemicals or treatments modulating the cellular metabolic capacity. To confirm the antiproliferative effects of the investigated compounds with a method independent of cell metabolism and redox reactions, we quantified total cellular biomass by crystal violet staining. The obtained results were in line with the data from the resazurin conversion assay (Table1 and Fig. 3B). To further assure that the decreased VSMC number upon treatment with test compounds is not due to cytotoxicity, we also quantified cell death by measuring LDH inside cells and in cell supernatants. No significant changes in cell viability were detected in the investigated concentration range (Table1 and Fig. 3C).

**Table 1:** Comparison of the activity of a ginger extract and several major pure compounds in VSMC and HUVECtert. Antiproliferative effects were evaluated by the resazurin metabolic conversion assay or crystal violet biomass staining. Cell death was evaluated by a colorimetric LDH activity assay as described in details in the “Materials and methods” section. The data shown represent means of three to five independent experiments each in triplicate or quadruplet. IC_50_ were determined by the GraphPad Prism software version 4.03 (GraphPad Software Inc., USA). ANOVA/Bonferroni analysis was used for evaluation of the statistical significance (n.s. indicates *p* > 0.05)

	VSMCs			HUVECs
	Resazurin metabolic	Crystal violet	Cell death	Resazurin metabolic
	conversion (IC_50_, μg/mL or μM)	biomass staining (IC_50_, μM)	(LDH release)	conversion
DCM extract	2.51 μg/mL	—	—	—
[6]-Gingerol	13.2 μM	8.77	n.s. (up to 100 μM)	n.s. (up to 30 μM)
[6]-Shogaol	2.72 μM	1.13	n.s. (up to 10 μM)	n.s. (up to 10 μM)
Zingerone	> 100 μM	—	—	—
[6]-Paradol	5.3 μM	3.34	n.s. (up to 30 μM)	n.s. (up to 30 μM)
*rac-*[6]-Dihydroparadol	10 μM	4.11	n.s. (up to 30 μM)	n.s. (up to 30 μM)

—: not detected.

n.s.: no significant difference.

DCM, dichloromethane.

**Figure 4:**
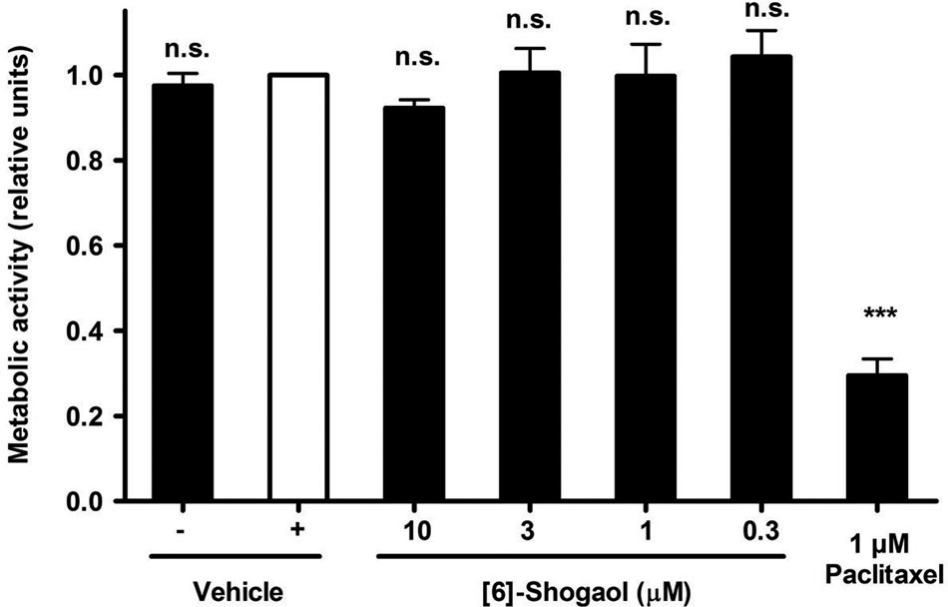
HUVECtert viability in the presence of [6]-shogaol. HUVECtert were treated with the indicated concentrations of [6]-shogaol or equal volume of the solvent vehicle (0.1% DMSO) for 48 h. Cell numbers were estimated at the end time point by the resazurin conversion method. All values are normalized to the signal obtained from vehicle (0.1% DMSO)-treated cells. The antiproliferative natural product paclitaxel (1 μM) was used as positive control. The data groups represent means ± SD from three independent experiments (n.s., not significant; ***p < 0.001; ANOVA/Bonferroni).

As vascular health also depends on a functional and intact endothelium, an optimal vasoprotective compound inhibits VSMC activation, but does not interfere with endothelial cell viability. We therefore investigated the impact of the four most active compounds on endothelial viability using metabolic activity as readout. In contrast to paclitaxel (1 μM), none of the four ginger compounds that clearly influence VSMC proliferation showed a negative effect on endothelial cells when used in the same concentration range, (Table1, Fig. 4). Interestingly, paclitaxel had an even stronger anti-proliferative effect in endothelial cells (IC_50_ = 4 nM) than in VSMC (IC_50_ = 108 nM; not shown). To assure that the observed differences in the potency of ginger compounds and paclitaxel in VSMC and endothelial cells are not due to different growth rates between the two cell types, we have determined the doubling times of the two cell types under the used experimental conditions. Cell counting revealed comparable proliferation rates for the PDGF-BB stimulated VSMC (cell doubling time 30.1 h) and the HUVECtert (cell doubling time 33.1 h).

Based on the observed promising antiproliferative profile of action on the VSMC, we have chosen the most potent identified compound, [6]-shogaol, for further mechanistic studies.

### 3.2 [6]-Shogaol leads to accumulation of VSMC in G_0_/G_1_

**Figure 5:**
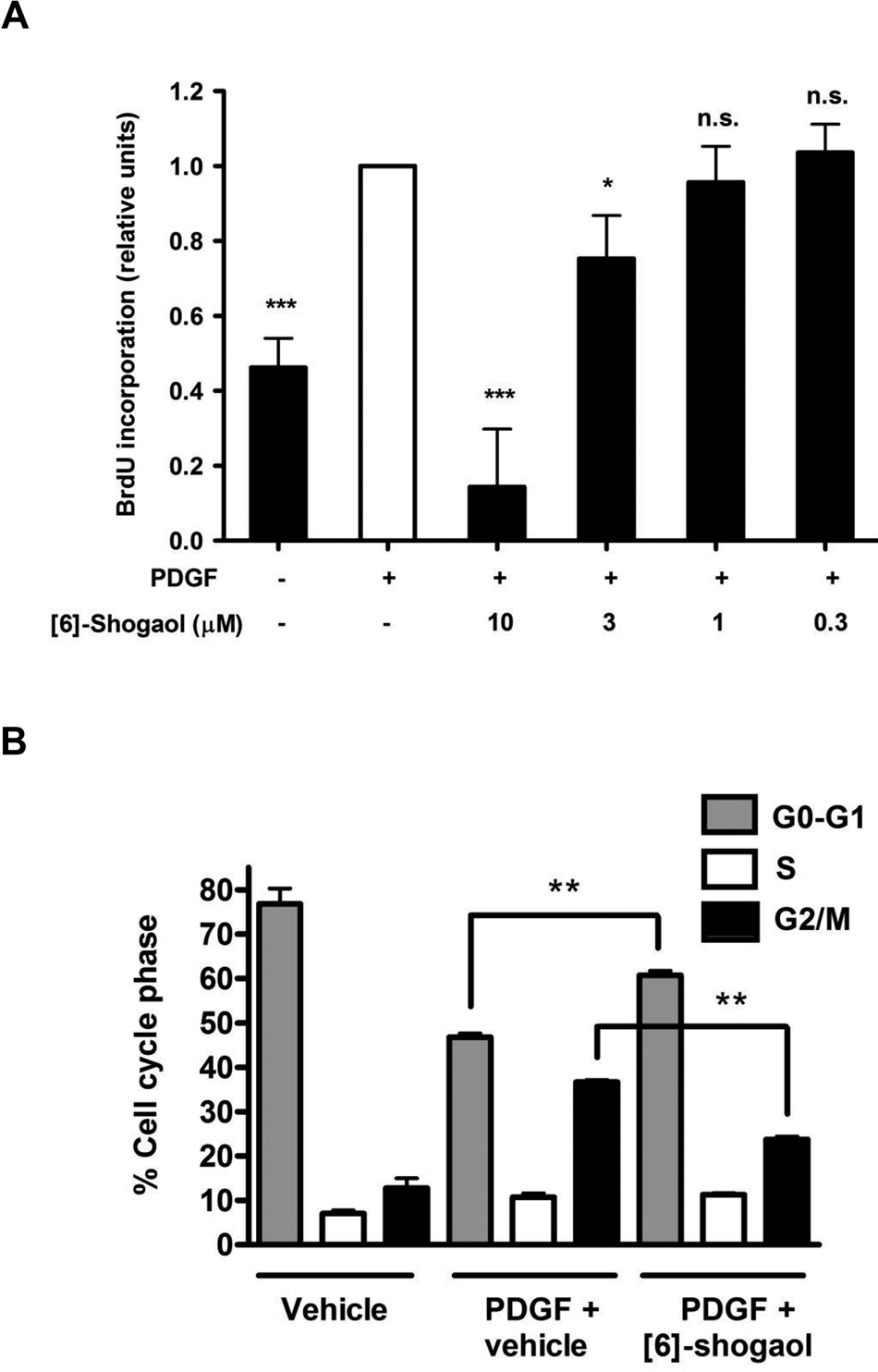
Effect of [6]-shogaol on cell-cycle distribution and DNA synthesis of VSMC. Quiescent VSMC were stimulated with 20 ng/mL PDGF-BB for 20 h, and cell proliferation was quantified by the BrdU incorporation in newly synthesized DNA in the presence of the indicated treatments (A); or the cells were incubated with [6]-shogaol (10 μM) or equal volume of the solvent vehicle (0.1% DMSO) for 30 min, then stimulated with 20 ng/mL PDGF-BB for 16 h, followed by PI nuclear staining and flow cytometry analysis (B). The data groups represent means ± SD from three independent experiments (n.s., not significant; ***p < 0.001; **p < 0.01; *p < 0.05; ANOVA/Bonferroni (A), 2-tailed paired *t*-test (B)).

In order to examine the mechanism of action of [6]-shogaol in VSMC, we first investigated whether it blocks DNA synthesis by quantification of BrdU incorporation in treated cells. [6]-shogaol potently blunted PDGF-stimulated DNA synthesis in a concentration-dependent manner exhibiting an IC_50_ of 3.0 μM, which is in the same range as the IC_50_ values obtained in the resazurin conversion and the crystal violet assay (Fig. 5A). In line with the observed inhibition of DNA synthesis, [6]-shogaol (10 μM) counteracted the PDGF-induced transition of VSMC from the G_0_/G_1_ cell-cycle phase to the G_2_/M phase, as revealed by flow cytometry analysis of PI stained nuclei (Fig. 5B).

### 3.3 [6]-Shogaol blocks VSMC proliferation by Nrf2-dependent HO-1 induction

**Figure 6:**
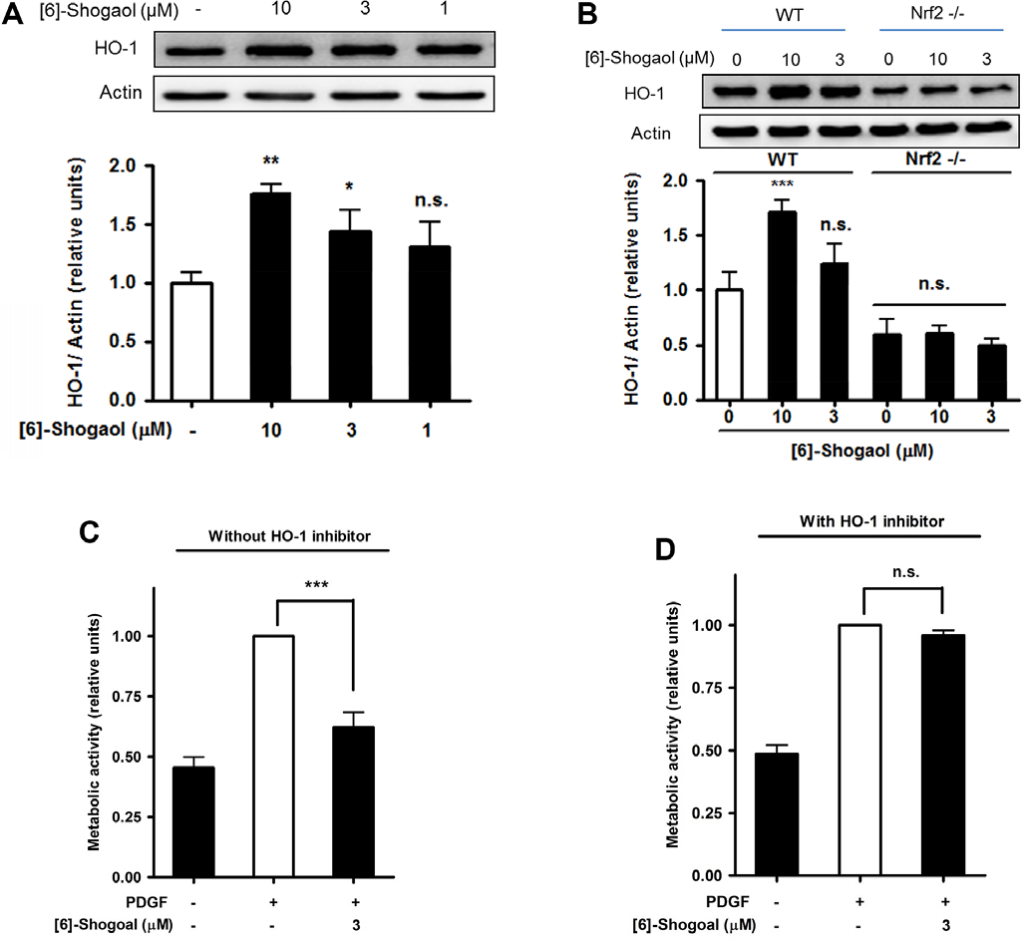
[6]-Shogaol induces HO-1 in Nrf2-dependent manner and HO-1 inhibition abolishes the antiproliferative effect of [6]-shogaol. (A) Quiescent VSMC were pretreated with the indicated concentrations of [6]-shogaol or an equal volume of the solvent vehicle (0.1% DMSO) for 30 min, and then stimulated with 20 ng/mL PDGF-BB for 20 h before their lysates were subjected to immunoblot analysis for HO-1 and actin as a loading control. Representative blots of three performed independent biological experiments (with one technical replicate) with consistent results are depicted. The bar graph shows compiled densitometric data of all performed experiments (mean ± SD, n.s., not significant; **p < 0.01; *p < 0.05; ANOVA/Bonferroni). (B) WT and Nrf2−/− mouse embryonic fibroblasts were treated with [6]-shogaol (10 or 3 μM) for 24 h before their lysates were subjected to immunoblot analysis for HO-1 and actin as a loading control. Representative blots are shown, the bar graph depicts compiled densitometric analyses from three biological replicates with one technical replicate each (mean ± SD, n.s., not significant; ***p < 0.001; ANOVA/Bonferroni). (C and D) Quiescent VSMC were pretreated with or without the HO-1 inhibitor SnPP (10 μM) for 1 h, then treated with [6]-shogaol (3 μM) or an equal volume of the solvent vehicle (0.1% DMSO) for 30 min, and then stimulated with 20 ng/mL PDGF-BB. Cell proliferation was determined by the resazurin conversion method. The data groups represent mean ± SD from three independent experiments (n.s., not significant; ***p < 0.001; ANOVA/Bonferroni).

In a consequent experiment the molecular events underlying the observed cell-cycle arrest in VSMC upon shogaol exposure were investigated. Among other bioactivities, [6]-shogaol is reported to be an activator of nuclear factor E2 related factor 2 (Nrf2) 24,25. Nrf2 is a transcription factor that is activated by a vast variety of stressors including oxidative insults, inadequate nutrient supply or electrophilic agents. Activated Nrf2 then launches a transcriptional program that mainly aims at detoxification and cellular stress resistance including cell-cycle control and metabolic adaptation (e.g. reviewed in 26,27). Activated Nrf2 and elevated expression and activity of the Nrf2-target gene HO-1 have been linked with reduced VSMC proliferation and prevention of cardiovascular disease 28–31. We therefore were prompted to analyse whether Nrf2 activation and subsequent HO-1 induction could possibly account for the growth arrest observed in shogaol-treated VSMC. For this, HO-1 expression in proliferating VSMC upon [6]-shogaol exposure was evaluated. Concentrations of 3 and 10 μM [6]-shogaol significantly elevated HO-1 levels compared to vehicle treated cells (Fig. 6A). Moreover, using WT and isogenic Nrf2-/- fibroblasts, a strictly Nrf2-dependent increase of HO-1 upon [6]-shogaol treatment (Fig. 6B) was observed, confirming Nrf2 activation by [6]-shogaol and excluding involvement of other transcription factors in the HO-1 induction in fibroblasts and most likely also in VSMC. In order to prove causality between the [6]-shogaol-induced HO-1 induction and proliferation stop in VSMC we made use of the HO-1 inhibitor tin protoporphyrin IX. As shown in Fig. 6C and D, [6]-shogaol loses its antiproliferative influence on VSMC in the presence of the HO-1 inhibitor. These findings indicate that [6]-shogaol induces HO-1 that then contributes to a reduced proliferation rate in VSMC.

## 4 Discussion

The present study reveals for the first time the inhibition of VSMC proliferation by a ginger extract and four major ginger constituents exhibiting IC_50_ values from 2.72 to 13.2 μM. [6]-shogaol, the most potent identified inhibitor, induces growth stop associated with accumulation of cells in G_0_/G_1_ and induction of HO-1 expression.

Ginger has been widely used as a culinary spice as well as in traditional oriental medicine for centuries. In recent years ginger attracted increasing attention due to a variety of newly described bioactivities promoting its use as a safe and effective medicinal plant 1,2,4,32. The nonvolatile pungent components, such as gingerols, shogaols, paradols, and zingerone, have been reported to be responsible for many of the reported biological effects of the plant 33–35. In fresh ginger, gingerols are the major pungent components, with [6]-gingerol being the most abundant. Gingerols are not stable, and their dehydration may yield large amounts of shogaols during prolonged ginger storage. Zingerone also cooccurs with shogaols in stored ginger. Shogaols may undergo reduction to form paradols that are also present in ginger. Shogaols are minor components in fresh ginger, while predominant pungent constituents in the dried ginger, with the ratio of [6]-shogaol to [6]-gingerol being around 1:1 in dried ginger 32–34,36,37. In commercially available ground ginger powders the amount of [6]-shogaol was reported to be in the range of 353–1459 μg/g 36,38. After a single oral administration of ginger oleoresin (300 mg/kg) to rats, the peak plasma concentration of [6]-shogaol was reported to be 0.11 μg/L (equalling 0.40 μM) 39. It is important to note that the anti-proliferative effect of [6]-shogaol on VSMC was observed at higher concentrations in our study, with the IC_50_s being 2.72 μM, 1.13 μM, and 3.0 μM upon quantification of metabolic activity (Fig. 3A), total biomass (Fig. 3B), and DNA synthesis (Fig. 5A), respectively. Therefore, although the here described anti-proliferative potential of [6]-shogaol might be of relevance upon local application (e.g. as a coating agent in drug-eluting stents), the question of whether acute or chronic oral administration of ginger could result in beneficial anti-restenotic effects requires further research.

Since all investigated ginger constituents are structurally related (Fig. 2), a comparison of their IC_50_ values (Table1) allows deducting a structure-activity relationship (SAR). All compounds possess a vanillyl moiety (4-hydroxy-3-methoxyphenyl) and an alkyl side chain, as shown in Fig. 2. They differ in the substitution pattern of the side chain at positions C3 or C5. [6]-shogaol, which was most active against PDGF-induced VSMC proliferation, possesses an α,β-unsaturated ketone at C3. [6]-paradol, only differing from [6]-shogaol by a saturation of the double bond, showed lower activity. [6]-gingerol, differing from [6]-paradol by having an additional hydroxy substituent at C5, showed even less potency. The activity of *rac*-[6]-dihydroparadol with a hydroxyl group at C3 was in a similar range as that of [6]-gingerol. Zingerone, also having a carbonyl group at C3, but a shortened and therefore less lipophilic alkyl side chain, showed little activity. Having all this in mind, the presence of an α,β-unsaturated ketone and the length of the alkyl side-chain seems to have significant impact on the extent of the observed antiproliferative activity. Interestingly, the investigated compounds showed a similar SAR when being investigated for anti-oxidative and anti-inflammatory effects 40, cytotoxicity and apoptosis induction in human promyelocytic leukemia (HL-60) cells 41, and protection of neuronal cells from β-amyloid insult 42.

Noteworthy, the Michael system of the α,β-unsaturated carbonyl type is involved in Nrf2 activation by [6]-shogaol and other small molecules 25,43. Moreover, HO-1 induction in VSMC by [6]-shogaol appears to be dependent on Nrf2 suggesting that the capacity of activating Nrf2/HO-1 is the basis for the observed SAR between the compounds.

Activation of Nrf2/HO-1 signaling also interferes with VSMC migration (e.g. 44) and cholesterol accumulation in macrophages (e.g. 45), as well as prevents endothelial dysfunction (e.g. 46) and inflammation (e.g. 47). These features make the hub an attractive target for the prevention of atherosclerosis by influencing different steps in the etiology of the disease. Analyzing whether [6]-shogaol could exert such pleiotropic vasoprotection by activation of Nrf2/HO-1 may therefore represent an interesting subject of future research. At this point it should be noted that although the causality between HO-1 induction and inhibited proliferation was demonstrated, it cannot be excluded that also other factors might be involved in the observed antiproliferative activity of [6]-shogaol. Likewise, inhibition of STAT3 or activation of PPARγ were reported for [6]-shogaol 48,49 and could also contribute to the cell-cycle arrest in VSMC 22,50.

Despite its potent effect on VSMC, [6]-shogaol did obviously not impair endothelial viability and proliferation (Fig. 4). Reendothelialization is a key step toward successful vascular healing after therapeutic interventions such as angioplasty or bypass surgery 51,52. Currently, the only clinical available treatment for restenosis is drug-eluting stent coated with rapamycin or paclitaxel 53. Both drugs not only inhibit VSMC, but also endothelial cells, causing impaired reendotheliazation and lengthening the healing of the wounded vessels 54,55. Our results on the apparent preferential activity of ginger compounds towards VSMC (Table1) may therefore inspire the future search for novel stent coating agents. Interestingly, [6]-shogaol exhibited its potent antiproliferation effect on VSMC without affecting the proliferation of endothelial HUVECtert cells.

In conclusion, the present study examines for the first time the inhibitory potential of ginger and its constituent [6]-shogaol towards VSMC proliferation. Furthermore, the structure-function relationship of several closely related ginger constituents is characterized and it is demonstrated that [6]-shogaol exerts its antiproliferative effect through accumulation of cells in G_0_/G_1_ associated with the activation of Nrf2/HO-1 pathway.

## Abbreviations

BrdU: bromodeoxyuridine
EBM: endothelial cell basal medium
FCS: fetal calf serum
HO-1: heme oxygenase-1
HUVEC: human umbilical vein endothelial cells
LDH: lactate dehydrogenase
MEF: mouse embryonic fibroblasts
Nrf2: nuclear factor-erythroid 2-related factor 2
PDGF: platelet-derived growth factors
PI: propidium iodide
SAR: structure-activity-relationship
VSMC: vascular smooth muscle cell
WT: wild type
